# [Retracted] miR‑218 functions as a tumor suppressor gene in cervical cancer

**DOI:** 10.3892/mmr.2024.13326

**Published:** 2024-09-09

**Authors:** Zhen Liu, Lin Mao, Linlin Wang, Hong Zhang, Xiaoxia Hu

Mol Med Rep 21: 209–219, 2020; DOI: 10.3892/mmr.2019.10809

Following the publication of this paper, it was drawn to the Editor's attention by a concerned reader that, concerning the Transwell cell migration and invasion assays shown in Fig. 4Ba and 4Ca on p. 215, quite a large number of the data panels contained overlapping sections, such that data panels which were intended to show the results from differently performed experiments had been derived from a smaller number of original sources.

After having considered this matter, in view of the number of overlapping data panels that were identified, the Editor of *Molecular Medicine Reports* has decided that this paper should be retracted from the Journal on account of a lack of confidence in the presented data. The authors were asked for an explanation to account for these concerns, but the Editorial Office did not receive a reply. The Editor apologizes to the readership for any inconvenience caused.

